# ISCB Ebola Award for Important Future Research on the Computational Biology of Ebola Virus

**DOI:** 10.1371/journal.pcbi.1004087

**Published:** 2015-01-29

**Authors:** Peter D. Karp, Bonnie Berger, Diane Kovats, Thomas Lengauer, Michal Linial, Pardis Sabeti, Winston Hide, Burkhard Rost

**Affiliations:** 1 International Society for Computational Biology, La Jolla, California, United States of America; 2 SRI International, Menlo Park, California, United States of America; 3 Department of Mathematics, Massachusetts Institute of Technology (MIT), Cambridge, Massachusetts, United States of America; 4 Computational Biology and Applied Algorithmics, Max Planck Institute for Informatics, Saarbruecken, Germany; 5 Hebrew University & Institute of Advanced Studies, Jerusalem, Israel; 6 Center for Systems Biology, Department of Organismic and Evolutionary Biology, Harvard University, Cambridge, Massachusetts, United States of America; 7 Department of Immunology and Infectious Disease, Harvard School of Public Health, Cambridge, Massachusetts, United States of America; 8 Broad Institute of Harvard and MIT, Cambridge, Massachusetts, United States of America; 9 Sheffield Institute for Translational Neuroscience, Harvard School of Public Health, Boston, Massachusetts, United States of America; 10 Department of Informatics, Bioinformatics & Computational Biology, TUM, Munich, Germany; 11 Department of Biochemistry & Molecular Biophysics, Columbia University, New York, New York, United States of America

## Abstract

Speed is of the essence in combating Ebola; thus, computational approaches should form a significant component of Ebola research. As for the development of any modern drug, computational biology is uniquely positioned to contribute through comparative analysis of the genome sequences of Ebola strains as well as 3-D protein modeling. Other computational approaches to Ebola may include large-scale docking studies of Ebola proteins with human proteins and with small-molecule libraries, computational modeling of the spread of the virus, computational mining of the Ebola literature, and creation of a curated Ebola database. Taken together, such computational efforts could significantly accelerate traditional scientific approaches. In recognition of the need for important and immediate solutions from the field of computational biology against Ebola, the International Society for Computational Biology (ISCB) announces a prize for an important computational advance in fighting the Ebola virus. ISCB will confer the ISCB Fight against Ebola Award, along with a prize of US$2,000, at its July 2016 annual meeting (ISCB Intelligent Systems for Molecular Biology [ISMB] 2016, Orlando, Florida).

## ISCB

ISCB, the International Society for Computational Biology, is dedicated to advancing the understanding of living systems through computation. ISCB now represents more than 3,200 computational biologists working in over 70 countries. It organizes more than seven annual international meetings and confers several major prizes, including the ISCB Senior Scientist Award, the ISCB Overton Prize, and the ISCB Outstanding Contributions Award. With the ISCB Fight against Ebola Award, the society offers for the first time an award for a specific scientific objective, thereby acknowledging the urgency of action required to fight a rising challenge.

## Ebola

The Ebola virus (EBOV) causes the Ebola virus disease (EVD), a disease with high fatality rates that has killed over 6,300 individuals from February to December 1, 2014 in the current outbreak [1]. The first reports assessing spreading risk have been published [2]. Recently, 99 EBOV genomes from 78 patients have been analyzed [3]. To facilitate global research, the respective author teams made all their data freely available. This laudable decision aligns with the ISCB open-access policy [4, 5]

Given the great urgency of creating an adequate response to Ebola in a short time frame, we urge funding agencies to develop special grant mechanisms for Ebola research, such as that which the United States National Science Foundation has recently announced [6]. For example, funding agencies could allow existing grant recipients to redirect funds from or apply for administrative supplements to appropriate existing grants for research dedicated to Ebola.

## ISCB Fight against Ebola Award

ISCB will confer the award, along with a prize of US$2,000, at the ISCB annual meeting, Intelligent Systems for Molecular Biology (ISMB), in July 2016, Orlando, Florida, US.

## Judgment Criteria

The award will be conferred on the submission that most closely meets the goal of providing an immediate solution from the field of computational biology as assessed according to the following terms: (1) high impact, (2) broad access, (3) measurable outcomes on understanding, handling, treating, or preventing the disease, and (4) close interaction with established mechanisms of Ebola control and research.

A selected team comprising experts chosen from areas such as Ebola research, epidemiology, public health, computational virology, structural biology, vaccine development, translational bioinformatics, genomics, and genome analysis will assess all submissions.

We will only consider submissions that remain within a limit of two pages. Please note that your submission could be in the style of a journal paper. Be advised of the following constraints when fitting your material onto the two pages: (1) the typeface must be Arial font throughout, using a minimum 9-pt font size for figures and a minimum of 10-pt Arial for the text. The precise minimum page margins depend on your choice of format (US-letter: a minimum of 1.25 inches on top and bottom and a minimum of 2.5 inches at the sides; A4: a minimum of 3 cm on top and bottom and a minimum of 1.5 cm on both sides.)

Submissions must be sent via email (a single PDF document) to Diane Kovats (admin@iscb.org) using the text “ISCB Fight against Ebola Award” in the subject and body of the email. The deadline for submission is April 10, 2016 (any time zone).
